# Bioactive Materials Based on Hydroxypropyl Methylcellulose and Silver Nanoparticles: Structural-Morphological Characterization and Antimicrobial Testing

**DOI:** 10.3390/polym15071625

**Published:** 2023-03-24

**Authors:** Anca Filimon, Mihaela Dorina Onofrei, Alexandra Bargan, Iuliana Stoica, Simona Dunca

**Affiliations:** 1 Polycondensation and Thermostable Polymers Department, “Petru Poni” Institute of Macromolecular Chemistry, Grigore Ghica Voda Alley 41A, 700487 Iasi, Romania; 2 Inorganic Polymers Department, “Petru Poni” Institute of Macromolecular Chemistry, Grigore Ghica Voda Alley 41A, 700487 Iasi, Romania; 3 Atomic Force Microscopy Laboratory, Physical Chemistry of Polymers Department, “Petru Poni” Institute of Macromolecular Chemistry, Grigore Ghica Voda Alley 41A, 700487 Iasi, Romania; 4 Department of Microbiology, Biology Faculty, “Alexandru Ioan Cuza” University of Iasi, 11 Carol I Bvd., 700506 Iasi, Romania

**Keywords:** hydroxypropyl methyl cellulose, silver nanoparticles, poly(N-vinylpyrrolidone), morphological characteristics, antimicrobial activity

## Abstract

The progress achieved in recent years in the biomedical field justifies the objective evaluation of new techniques and materials obtained by using silver in different forms as metallic silver, silver salts, and nanoparticles. Thus, the antibacterial, antiviral, antifungal, antioxidant, and anti-inflammatory activity of silver nanoparticles (AgNPs) confers to newly obtained materials characteristics that make them ideal candidates in a wide spectrum of applications. In the present study, the use of hydroxypropyl methyl cellulose (HPMC) in the new formulation, by embedding AgNPs with antibacterial activity, using poly(N-vinylpyrrolidone) (PVP) as a stabilizing agent was investigated. AgNPs were incorporated in HPMC solutions, by thermal reduction of silver ions to silver nanoparticles, using PVP as a stabilizer; a technique that ensures the efficiency and selectivity of the obtained materials. The rheological properties, morphology, in vitro antimicrobial activity, and stability/catching of Ag nanoparticles in resulting HPMC/PVP-AgNPs materials were evaluated. The obtained rheological parameters highlight the multifunctional roles of PVP, focusing on the stabilizing effect of new formulations but also the optimization of some properties of the studied materials. The silver amount was quantified using the spectroscopy techniques (energy-dispersive X-ray fluorescence (XRF), energy-dispersive X-ray spectroscopy (EDX)), while formation of the AgNPs was confirmed using Fourier transform infrared spectroscopy (FTIR), X-ray diffraction (XRD), transmission electron microscopy (TEM), and dynamic light scattering (DLS). Also, the morphological examination (Atomic Force Microscopy (AFM) and Scanning electron microscopy (SEM)) by means of the texture roughness parameters has evidenced favorable characteristics for targeted applications. Antibacterial activity was tested against *Escherichia coli* and *Staphylococcus aureus* and was found to be substantially improved was silver was added in the studied systems.

## 1. Introduction

The medical field is faced with various infectious diseases triggered by germs (bacteria, viruses, fungi and protozoa), associated with microbial resistance. This is the main reason for the current intense research into antibacterial agents. In addition, microorganisms which contaminate the surfaces of medical devices in hospitals have led to development of antimicrobial surfaces based on polymers as a necessary tool to combat microbial infections and prevent the spread of microbial cells [1]. In this context, polymeric materials, due to their intrinsic properties, e.g., their hydrophilicity or molecular weight, have gained an increasing interest from both academic and industrial perspectives [2,3,4,5,6,7,8,9]. This is because they can determine the final antimicrobial activity in terms of the biocide release rate, as a result of their synergistic activities [4,10,11]. Additionally, the polymers can act as a matrix for the materials holding antimicrobial agents, thus leading to the development of new materials, which are both bactericidal and biocompatible. Moreover, antimicrobial polymers usually have a longer-term activity [12,13,14,15,16]. Thus, systems based on the combination of polymer and inorganic/organic antimicrobial agents present a special interest. In this regard, the improvement of the antimicrobial activity and the reduction in the toxicity will be ensured by the incorporation of the antimicrobial agent. Based on the various studies presented in the literature [8,17,18,19,20], a small amount of active component, less than 5 wt.%, has been identified as conferring an efficiency to the whole system. In addition, the investigation of inexpensive systems through different approaches aimed at combating infections caused by microorganisms, will be pursued.

Cellulose-based materials are important for this purpose due to the properties they possess, such as biocompatibility, barrier properties, non-polluting, lack of toxicity, and low-cost [21]. However, the cellulose derivatives, as result of the chemical modifications, tend to improve the cellulose backbone in terms of solubility, viscosity, and film-forming performance [22,23,24]. Notably, hydroxypropyl methyl cellulose, HPMC, is an attractive polymer that can be used for wider applications in the biomedical field because it is a readily available non-ionic plant derivative that forms transparent, flexible, water-soluble films with efficiency in oxygen and carbon dioxide transport [25,26,27,28]. All these characteristics justify its good applicability for medical purposes; the progress focusing on selectivity and increasing its durability.

The development of new antimicrobial polymeric materials containing metal ions with a therapeutic or diagnostic impact is undeniable. In these conditions, the newly obtained compounds act through a different mechanism than the known ones for which many pathogens are more resistant, which includes compliance with the principles of infection control using the new strategy to be addressed. In conclusion, expanding the application of polymeric materials in several fields involves the use of metal and/or metal oxide nanoparticles, thus improving their antimicrobial activity [29,30,31]. Thus, to improve their antimicrobial activity, the cellulose matrix requires the interaction with metals. Cellulose by itself is insoluble and therefore cannot easily be used for the generation of nanoparticles. Comparatively, HPMC is a soluble cellulose derivative which has been tested as a reducing and stabilizing agent [32,33,34]. Nevertheless, even under these conditions, there are various suitable additives that lead to the improvement of HPMC properties in terms of functional performance and/or enhanced properties [35].

Due to its bactericidal effect, silver has been intensively studied in comparison with other antimicrobial agents to prevent and control infections. Currently, the impact of silver nanoparticles, as a result of their multifunctional properties [36], is being evaluated as a component of bioactive materials by combining its antibacterial property with the particular performance of the used biomaterial [37,38]. Furthermore, various studies have confirmed the oligodynamic quality of Silver and its therapeutic effect against a wide range of microorganisms (over 650 species) [12,13,39,40].

To obtain a better dispersion and fixation/catching of AgNPs in the HPMC matrix, the poly(N-vinylpyrrolidone) (PVP) has attracted considerable attention because it acts not only as a stabilizing agent but also has an impact on the control of the reduction rate of the silver ions and the aggregation process of silver atoms [41,42]. It is known to have excellent chemical and physical properties that make it a good material as additive or coating to other materials, including HPMC [43,44,45,46]. Thus, the utilization of PVP as a stabilizer facilitates the binding of the metal, leading to the improvement of the biomedical properties. As result of PVP usage, cellulose materials interact with metals at the target site or disturb the balance of metal–ion uptake and distribution in cells and tissue. The understanding of these interactions leads to a rational design of metal-polymer systems and the implementation of new co-therapies [47,48,49]. Therefore, the antimicrobial functions of the novel materials based on HPMC can be considered as a potential tool in reducing disease transmission, assuming that they could reduce the microorganism’s population. 

Based on the above, the urgent need to control infectious diseases has led to the formulation of a strategy with the aim of obtaining new cellulosic biomaterials based on inorganic active agents with bactericidal effect. Therefore, the main purpose of this paper is to study the functionality and efficiency of new materials based on HPMC and AgNPs and to analyze the possibility of their use as antibacterial materials; and biomaterials. Thus, the use of HPMC/PVP-AgNPs materials avoids some shortcomings and combines the properties of the used polymers, and through the applied technique ensures the efficiency and selectivity of the obtained materials.

## 2. Materials and Methods

Hydroxypropyl methyl cellulose (HPMC), poly(N-vinylpyrrolidone) (PVP)—as stabilizing agent (M_w_ = 10,000 g/mol) and silver nitrate (AgNO_3_)—as the metal precursor (M_w_ = 169.88 g/mol) were purchased from Sigma-Aldrich Company (Darmstadt, Germany) and used as received, without further purification. All the experimental study was performed in double distilled water. Antibacterial testing was carried out on Gram-positive *Staphylococcus aureus* (ATCC 25923) and Gram-negative *Escherichia coli* (ATCC 25922) bacteria.

The homogeneous solution of HPMC, with a concentration of 10 g/dL, was prepared by dissolution in double-distilled water and kept for one week to swell until complete dissolution was achieved, under gentle heating in a water bath at 50–55 °C and 600 rpm. The solution was cooled gently to room temperature and then kept until use. Afterwards, a solution of PVP having the same concentration of 10 g/dL in double-distilled water was made ready by stirring (600 rpm) in a water bath (50 °C) until the PVP had completely dissolved. Both solutions were mixed at different amounts, preparing HPMC/PVP blends with varied compositions (i.e., 95/5, 90/10, 85/15, 80/20, 75/25, 70/30 (wt.%/wt.%)). To prevent the formation of voids inside the membrane, the samples were allowed to degas for 24 h at ambient conditions. Subsequently, the solutions were cast on a Teflon substrate and after casting were solidified, initially by slow drying in saturated atmosphere of the used solvent. Thus, we obtained polymer films that were considered definitive when easily removed from the Teflon support.

According to the protocols presented in the literature [50,51,52], the incorporation of silver nanoparticles in the aqueous solutions containing HPMC by thermal reduction of silver ions to silver nanoparticles (using PVP as a stabilizer) was performed. Therefore, different concentrations of AgNO_3_ were added to aqueous solutions containing HPMC, PVP, and 70/30 wt.%/wt.% composition of HPMC/PVP blend, finally keeping only the concentration of 0.05 mg/mL for the study. The solutions were maintained under continuous stirring for 4 h at 70–80 °C at a speed of 650 rpm, to attain homogeneous solutions. Subsequently, the contents were cooled and centrifuged for 30 min. During agitation, the silver ions were stabilized in the solution; the metal ions being connected to the functional groups of the HPMC and PVP networks. At the end, a change in color from transparent—reddish to brown was observed, demonstrating the formation of silver nanoparticles (AgNPs). The solutions were stable for 1 month [50]. The prepared solutions were cast on a Teflon substrate, as mentioned in the protocol for obtaining silver-free films.

The rheological behavior of HPMC, PVP and their blends in double-distilled water was performed using a Bohlin CS50 rheometer (Malvern Instruments, Worcestershire, UK) with cone-plate geometry (cone angle of 4° and 40 mm diameter). Dynamic viscosities were recorded over the range of shear rates varying between 0.1–100 s^−1^ and 25 °C temperature with an accuracy of ± 5%. NTEGRA Scanning Probe Microscope from NT-MDT Spectrum Instruments with an NSG03 cantilever (both from NT-MDT Spectrum Instruments, Zelenograd, Moscow, Russia) was used to investigate the morphological modifications induced on the surface of the samples HPMC, PVP and HPMC/PVP by the incorporation of the Ag nanoparticles. The atomic force microscopy technique (AFM) was applied in semicontact mode, in atmospheric conditions, at room temperature. The resonance frequency of the cantilever was 93 kHz. The scanning frequency was 0.6 Hz. The AFM images were registered and analyzed using the Nova 1.0.26.1443 and Image Analysis 3.5.0.19892 software (NT-MDT Spectrum Instruments, Zelenograd, Russia).

Surface morphology and elemental composition for the obtained samples containing pure polymers (HPMC, PVP) and their blends with AgNO_3_ were performed using a Verios G4 UC Scanning electron microscope (Thermo Scientific, Prague, Czech Republic) equipped with Energy Dispersive X-ray spectroscopy analyzer (Octane Elect Super SDD detector, FEI Company, Hillsboro, OR, USA). Scanning Electron Microscopy (SEM) analyses were performed using a backscatter electron detector (Angular Backscattered Detector, ABS, Delong Instruments, Quebec, QC, Canada) with 5 kV accelerating voltage, in order to obtain compositional contrast images.

To evaluate the specific surface area of obtained materials, the sorption and desorption isotherms were registered using a Dynamic Vapor Sorption (DVS) Analyzer (IGAsorp, Hiden Analytical, Warrington, UK). Detailed procedure of the experiment has been presented in a previous study [7]. The energy-dispersive X-ray fluorescence (XRF) system (EX-2600 X-Calibur SDD, Malver Panaalytical Ltd., Grovewood, UK) was used to confirm the presence of metal (Ag) in the structure of the materials. Fourier transform infrared (FTIR) spectra were recorded on a Bruker Vertex 70 spectrometer (Bruker Optics, Ettling, Germania) in the 4000–600 cm^−1^ region to detect the functional groups involved in the formation of silver nanoparticles by the reduction of silver nitrate. In addition, the X-ray diffraction (XRD) studies on phase composition for materials obtained based on HPMC with embedded silver were performed on a Rigaku Miniflex 600 diffractometer (Riga, Latvia) using CuKα-emission in the angular range 3°–90° (2θ) with a scanning step of 0.01° and a recording rate of 2°/min at room temperature.

Transmission electron microscopy (TEM) and dynamic light scattering (DLS) were applied to evaluate the distribution and size AgNPs, as well as their polydispersity index (PDI). The TEM analysis of the samples was carried out using a Hitachi HT7700 (Tokyo, Japan) instrument, operating in contrast mode at 100 kV. DLS analyses were performed on a Malvern Nano-ZS device (Malvern Instruments, Worcestershire, UK) at 25 °C. For each sample the average of at least three measurements was used. Data analysis was realized with the Zetasizer software provided by Malvern (Grovewood, UK) and the graphs with Origin 2018 SR. The in vitro antimicrobial activity was performed by the disc diffusion method (Kirby-Bauer) according to the Clinical and Laboratory Standards Institute (CLSI). The culture medium (Mueller-Hinton agar) was distributed in Petri dishes (20 mL). The bacterial strains tested (*Staphylococcus aureus* (*S. aureus*) and *Escherichia coli* (*E. coli*)) were cultivated in a Mueller-Hinton Broth medium at 70 °C, 24 h, the bacterial inoculum was obtained and adjusted to a density corresponding to the 0.5 McFarland standard (1.5 × 10^8^ CFU). The bacterial suspension was uniformly distributed over the entire surface of the culture medium. The analyzed films (HPMC, HPMC-AgNPs, 70/30 wt.%/wt.% composition of HPMC/PVP, namely HPMC/PVP, and HPMC/PVP-AgNPs) were cut in the shape of a disc (10 mm in diameter) and applied to the culture medium surface. After incubation at 37 °C for 24 h, the zone size of inhibition was measured using a graduated ruler.

## 3. Results and Discussion

### 3.1. Conformational Characteristics in the HPMC/PVP/Water System: Rheological Parameters

The achievement of polymer materials with resistance to the mutations of microbial organisms—antimicrobial polymeric systems—involves a first stage where the control of the properties in solution, dictated by the configuration and conformation of the polymeric chains, which influence their final arrangement/organization in the solid state and implicitly, are assigned the properties of the final material desired for the application. Thus, the formation process of the antimicrobial polymeric systems that could be used in requires taking into account both the properties that determine the orientation of the polymer chains established by the different composition of the bulk components, as well as the biological ones. The previous viscometric study performed on HPMC/PVP dilute solutions [53] showed, based on the obtained results from the evaluation of the solubility parameters and the intrinsic viscosity, a good relationship between the solubility properties and structural-conformational characteristics of the two components in the system. For this reason, and as a result of the optimal molecular aspects of this polymer mixture, previously obtained, the present study aimed to establish the specific characteristics that recommend this polymer mixture for practical applications. Considering the composition of the system, as well as the proposed methodology, we expected this study to reveal some additional information that was not previously known.

**Figure 1:**
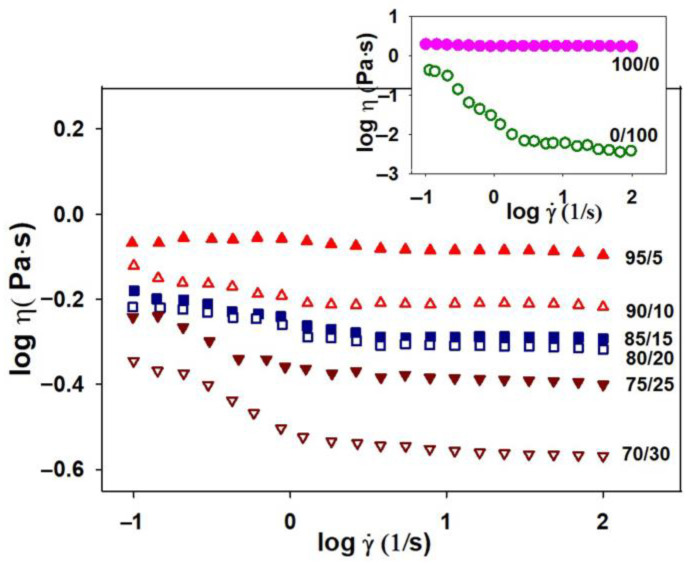
Rheological profile illustrated by double-logarithmic plots of dynamic viscosity (η) vs. shear rate (γ˙) for pure HPMC, PVP (small plot) and HPMC/PVP studied systems at various mixing ratios and 25 °C.

In this context, the knowledge of rheological parameters was important because they have an influence on the polymer solutions processing and, implicitly, on the final product properties. Thus, the rheological behavior of HPMC, PVP and their mixtures in different mixing ratios, in double-distilled water, is a complex one and can be evaluated from the variation of the dynamic viscosity based on the shear rate (Figure 1).

As can be observed, the Newtonian regime appears for pure HPMC and 95/5 wt.%/wt.% composition of the HPMC/PVP blend for whole studied shear rate domain. Instead, as the content of HPMC decreases (i.e., 90/10, 85/15 and 80/20 wt.%/wt.% compositions of the HPMC/PVP blends) a non-Newtonian behavior, thinning behavior, begins to be visible at low shear rates (Region I), followed by a large Newtonian region (Region II), as shear rate increases. On the other hand, it is observed that, as the PVP content increases, exceeding 20 wt.% (i.e., 75/25 and 70/30 wt.%/wt.% composition of the HPMC/PVP blends), there is a change in flow behavior, the thinning behavior is predominantly even for low shear rates, and the Newtonian plateau decreases.

The non-Newtonian behavior can be explained [54,55] as a result of the tendency of polymer chains to elongate in the shear direction. As the shear force increases, becoming predominant, the polymer chains orient in the flow direction. Thus, as a consequence of the changes in the material structure, resulting from the interactions between the polymer chains and dispersion medium, a thinning behavior appears. In addition, as result of the hydrophilic nature, PVP has the ability to form hydrogen bonds with the solvent (water) [56], so that in aqueous solution it can exist in an entangled form without forming a compact gel network. In these conditions, the shear flow favors the intra-chain associations, leading to a decrease in viscosity [54]. Hence, the addition of PVP in the system will lead to an intensification of the physical and chemical interactions, thereby having impact on the rheological characteristics of the systems. As the PVP ratio in the blend increased from 5 to 30 wt.%, the blends exhibited an enhancement of the rheological properties, revealing the role of PVP as a polymeric stabilizer, due to the strong polarity and hydrophilic properties [57,58]. Consequently, the different and complex rheological behavior can be explained by taking into account the synergism between the structural characteristics of both HPMC and PVP, which in turn depends on the packing efficiency and specific interactions.

On the other hand, the rheological behavior illustrated in Figure 1 is described by the values of the flow behavior (n) and consistency (k) indices, evaluated according to the Ostwald-de Waele model (Equation (1)).

(1) $$\sigma =k\times {\gamma ˙}^{n}$$

**Figure 2:**
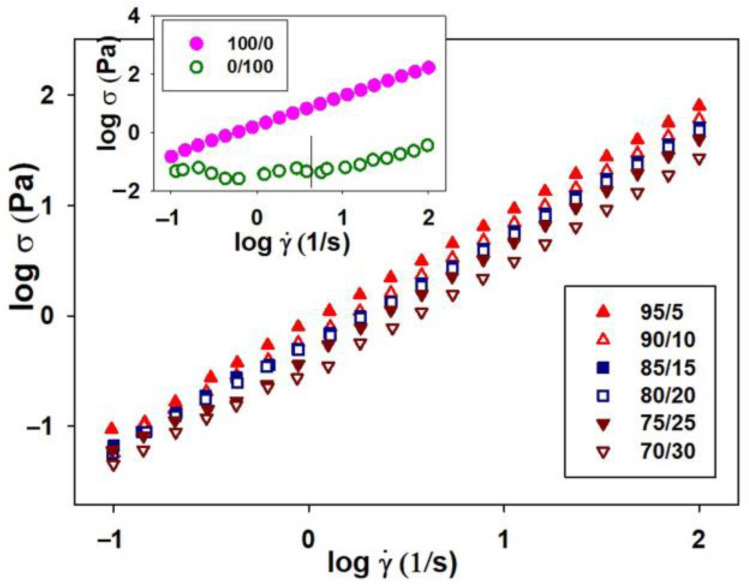
Double-logarithmic plots of shear stress (σ) vs. shear rate (γ˙) for pure samples HPMC, PVP (small plot), and HPMC/PVP studied systems in different mixing ratios at 25 °C.

According to the data represented in Figure 2, the exponents of the power law vary function on PVP content from the studied blends (see Table 1).

Thus, the flow behavior indices were close to the unit for HPMC and blends that dis not exceed 15 wt.% (e.g., 95/5, 90/10, and 85/15 wt.%/wt.% compositions), highlighting the Newtonian behavior, in accordance with the rheological profiles illustrated in Figure 1. Instead, the flow curve for pure PVP exhibited two distinguished regions, characterized by different slopes (Figure 2, small graph), where the power law index, n, takes values lower than unity (Table 1), revealing a shear thinning (pseudoplastic) behavior.

Conforming to literature data [59], when n ≅ 1, the fluid has a Newtonian behavior, while n < 1 or n > 1 the fluid presents a thinning or thickening behavior, respectively. In agreement with this statement, the obtained data presented in Table 1 confirm the thinning behavior as a result of PVP being added into the blend; this aspect was indicated by the sub-unitary values of flow indices. Any increase in PVP content was manifested by the slight decrease in the values of the flow behavior index. On the other hand, as PVP was added into the system, the values of consistency indices gradually decreased for all compositions of the HPMC/PVP blend.

Consequently, through the obtained rheological profiles/parameters the multifunctional roles of PVP were highlighted, focusing on the effect of PVP in terms of physical stabilization of various formulations in solid state but also the optimization of several properties of the studied blends. Based on all mentioned aspects, the PVP was added to HPMC solution to ensure an optimal composition (70/30 wt.%/wt.% composition of HPMC/PVP blend) that favored a good stabilization of silver nanoparticles (AgNPs), thus assuring specific properties, e.g., a high antimicrobial activity suitable for biomedical applications.

### 3.2. New Formulations by Silver Introduction in HPMC/PVP System

Metal complexes have the advantage of developing new materials with an antibacterial activity. They are easily adaptable to the synthesis conditions and, as a consequence, can result in a wide structural variety. Metallic centers have the ability to organize surrounding atoms in different geometries which are difficult to obtain through other ways. Moreover, the specific effects of metals can be adapted by recruiting cellular processes involving metal-macromolecules interactions [60]. For silver to be used as a bactericidal agent, according to studies in the field [61], the oxidation to the Ag^+^ ion is necessary. The process is slow and the effective silver concentration is low. The synthesis process involves the reduction of soluble silver salts with different reducing agents both in water and in organic solvents [62]. In order to avoid obtaining large silver nanoparticlesor aggregates, stabilizing compounds can be used. The initially formed silver metallic nuclei can have different sizes and shapes because of variation in the synthesis conditions (concentration, reduction agent, temperature, presence of additives) [62,63]. According to studies in the field [64], the poly(N-vinylpyrrolidone) (PVP) and substituted phosphane ligands can be successfully used as stabilizers. Generally, relying of its special physico-chemical and biological properties, poly(N-vinylpyrrolidone) is suitable and promising for obtaining different materials for medical applications [50,65,66]. On the other hand, the addition of natural and synthetic polymers as stabilizers, through different methods, aims to obtain AgNPs with well-controlled sizes and shapes, and at the same time prevent agglomeration and nanoparticle coalescence [67,68].

**Figure 3:**
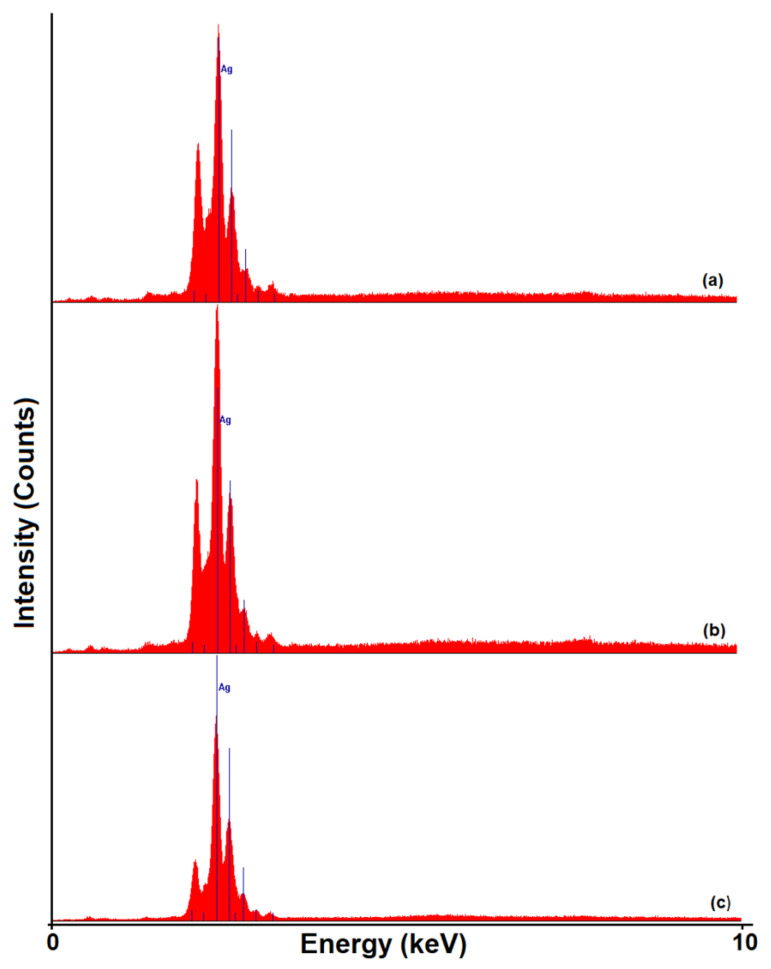
XRF spectra of the samples: HPMC-AgNPs (**a**), PVP-AgNPs (**b**), 70 HPMC/30 PVP-AgNPs (**c**).

Based on the above statements, the preparation of antimicrobial systems based on HPMC with high inhibiting effect on the microbial growth were obtained using the thermal reduction method using PVP which helps to control the size and distribution of silver nanoparticles and prevent their precipitation. In particular, the nondestructive analysis method, the energy-dispersive X-ray fluorescence (XRF), was employed to detect the Ag presence in the newly obtained formulations (Figure 3). The induced changes in the achieved materials as result of the nanoparticle formation were evaluated by the intensity of silver characteristic peaks. Thus, it was possible to demonstrate how the AgNPs were distributed in the polymeric matrix, depending on the used solvent and how they were stabilized in the films structure.

**Figure 4:**
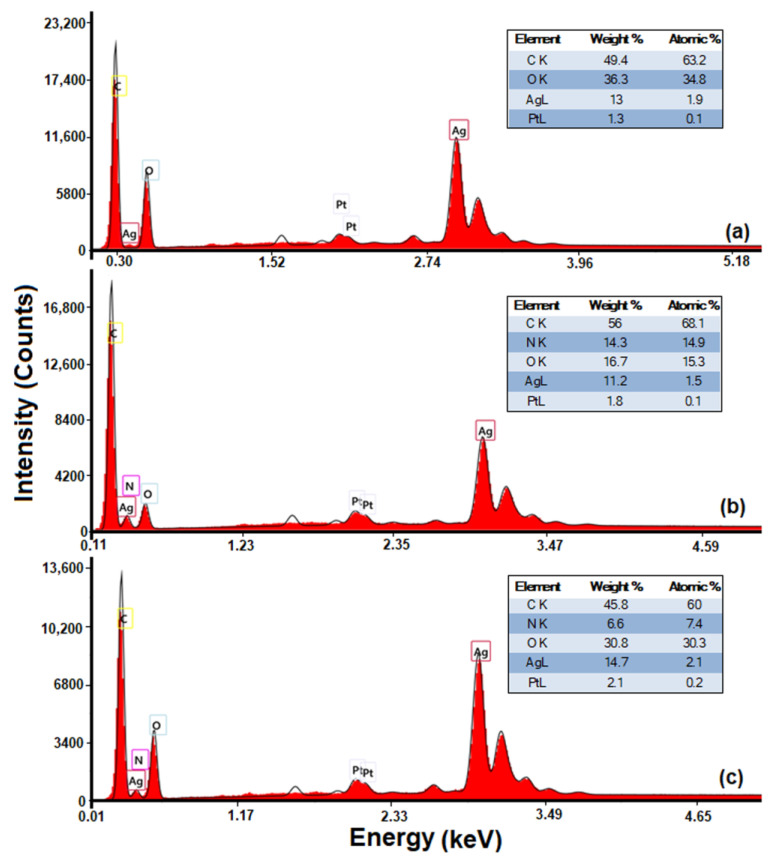
EDX spectra for: HPMC-AgNPs (**a**), PVP-AgNPs (**b**), 70 HPMC/30 PVP-AgNPs (**c**).

Similar to the XRF data, the enrichment of the polymeric matrix with silver was observed by analysis of the chemical structure and of all the elements identified in the EDX spectra (Figure 4). In accordance with the listed results in Figure 4, the values of silver content in the polymeric matrix represent an indication that PVP has an impact on silver nanoparticles distribution, preventing their agglomeration and/or precipitation. Moreover, the stabilization tendency of AgNPs during the preparation process observed from the values obtained for the peak’s intensity (Figure 3) was also confirmed by the values of the silver content (Figure 4).

It is known that the stabilization of nanoparticles can be achieved either with steric (by polymer doping) or electrostatic forces (which involve surface modifiers) [69]. Accordingly, in the HPMC-AgNPs complex, the nanoparticles were stabilized only sterically, this could be expected because there was no covalent bond between the HPMC and Ag [70]. Instead, the AgNPs stabilization in PVP-AgNPs and HPMC/PVP-AgNPs complexes was determined by the catching agent due to the local polar moment’s effects influencing the nanoparticles surface [70,71]. The catching mechanism can be explained by taking into account the hydrophilic nature of the amide groups and the hydrophobic nature of the vinyl groups of the PVP. As result of the strong affinity between N and O atoms for transition metals, AgNPs will bind to the amide groups of PVP. In addition, due to the hydrophobic characteristic, the vinyl backbone that will surround the AgNPs will prevent their aggregations [72,73]. This behavior demonstrates that by adding PVP, the peak intensity and silver content from the polymeric blend increased. These results confirmed a better stabilization of AgNPs in a polymeric matrix.

**Figure 5:**
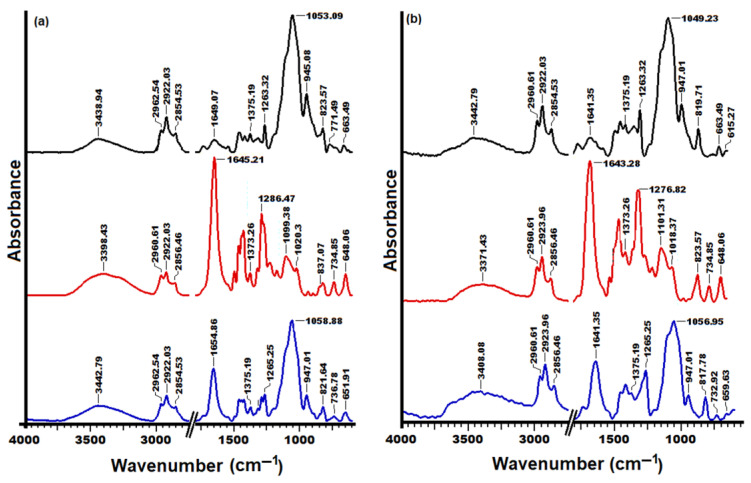
FTIR spectra of free (**a**) and embedded with AgNPs (**b**) HPMC (black), PVP (red), 70 HPMC/30 PVP (blue) samples.

Additionally, in order to confirm the hypothesis for the formation of silver nanoparticles in the studied materials by the reduction of silver nitrate as a result of the functionality of HPMC and PVP molecules, FTIR spectroscopy was carried out. In this sense, FTIR spectra of the pure and embedded AgNP samples were compared (Figure 5).

According to the literature [74,75], FTIR spectra should exhibit several bands that are typical of HPMC and PVP structures, namely: the broad transmission band at 3100–3600 cm^−1^ attributed to the stretching vibrations of the –OH groups; the absorption peaks between 2850 cm^−1^ and 2960 cm^−1^ due to the asymmetric and symmetric CH_2_ stretching and the peaks in the range 1240–1500 cm^−1^ which assigned to the C–H bending vibration; the stretching vibrations of C–O–C asymmetric and the C–O groups observed at 1060 cm^−1^ and 1100 cm^−1^. Additionally, the characteristic band of the amide carbonyl stretch from PVP is positioned around 1650 cm^−1^ and also, the vibration peak of propyl stretching, from HPMC, which should reach a maximum at approximatively 1650 cm^−1^.

Comparing the FTIR spectra of the studied samples (Figure 5a,b), changes occur associated with the displacement of the bands from about 3400 cm^−1^ and 1640 cm^−1^, respectively. This result indicates functionality reactive groups of the studied polymeric materials in the process of reducing silver nitrate, confirming their ability to form nanoparticles. On the other hand, according to the literature [75], the band at approximately 1380 cm^−1^ indicates the presence of silver nitrate. According to the data recorded in the FTIR spectra, the appearance of a weak peak or its absence represented another indicator that highlighted the functionality of the groups as a result of the reduction of silver nitrate.

**Figure 6:**
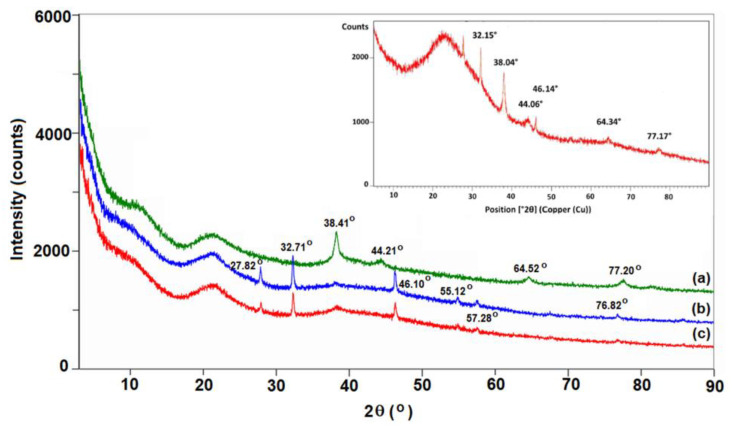
XRD spectrum of studied materials: (**a**) PVP-AgNPs, (**b**) HPMC-AgNPs, (**c**) 70 HPMC/30 PVP-AgNPs. Inserted graph represents the XRD pattern of AgNPs according to the standard ICSD No. 98-018-0878.

The structural features related to the phase composition for the materials based on HPMC with embedded silver were performed using XRD measurements. The identified distinct diffraction peaks according to Figure 6 revealed the crystalline nature of the synthesized particles. Moreover, the obtained experimental results were found to be inconsistent with those reported for the diffraction pattern of AgNP [76]. Additionally, the peaks near to 27.8°, 32.7°, 46.1°, 55.1°, 57.2° may have been due to the presence of a polymeric phase on the surface of particles. Based on the obtained results, the ability of PVP to stabilize the silver nanoparticles more strongly than the cellulosic derivative was confirmed, in accordance with the XRD pattern of ICSD standard No. 98-018-0878.

**Figure 7:**
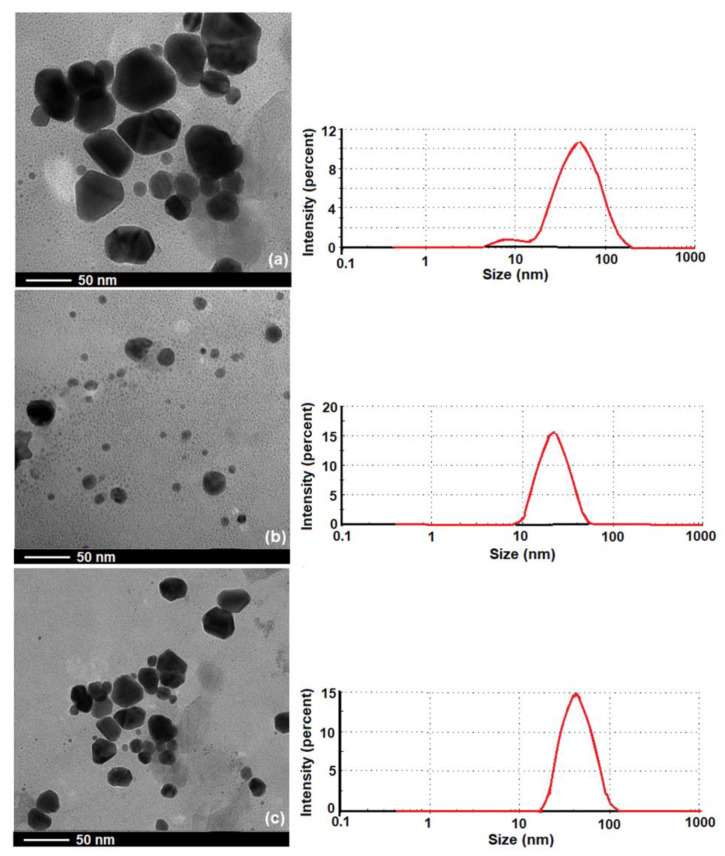
TEM micrographs and DLS particle size distribution graph of the studied materials: (**a**) HPMC-AgNPs, (**b**) PVP-AgNPs, (**c**) 70 HPMC/30 PVP-AgNPs.

The distribution and size of the AgNPs in the studied polymeric material matrix (HPMC, PVP, and their blend) were evaluated by TEM micrograph (Figure 7). TEM images showed that the AgNPs were well-dispersed without large agglomeration in the case of the PVP and HPMC/PVP matrices, having predominantly spherical or globoid shape with various diameters (Figure 7b,c). Instead, in the case of the HPMC matrix, the morphological structure of AgNPs indicated different sizes and shapes, some being in aggregated form, loosely distributed in the polymer matrix (Figure 7a).

The average particle size distribution of AgNPs was measured with DLS (Z-average) as shown in Figure 7. The obtained results were compared to those obtained from TEM and are listed in Table 2. The observed differences between the obtained parameters can be attributed to the applied methods, namely DLS depends on the hydrodynamic size, while TEM depends on the physical size. Furthermore, the sizes observed by DLS were larger than the results of the TEM images, which was due to the screening of small particles by large ones [76,77]; an aspect which also confirms the polydispersity of AgNPs.

According to the obtained data, the size of the silver nanoparticles in the HPMC matrix was larger and they tended to agglomerate; in addition, the size distribution was wider compared to that of the silver nanoparticles in the PVP matrix, indications that confirm the maintenance of better stability. Therefore, the choice of the stabilizing agent was very important from the perspective of the possibility of providing a long-term anti-infection effect [12]. So, the obtained outcomes have demonstrated that the achieved materials potentiate their functionality, sustainability, and performance, minimizing the bacterial cells adhesion and proliferation.

### 3.3. Surface Morphology Analysis

**Figure 8:**
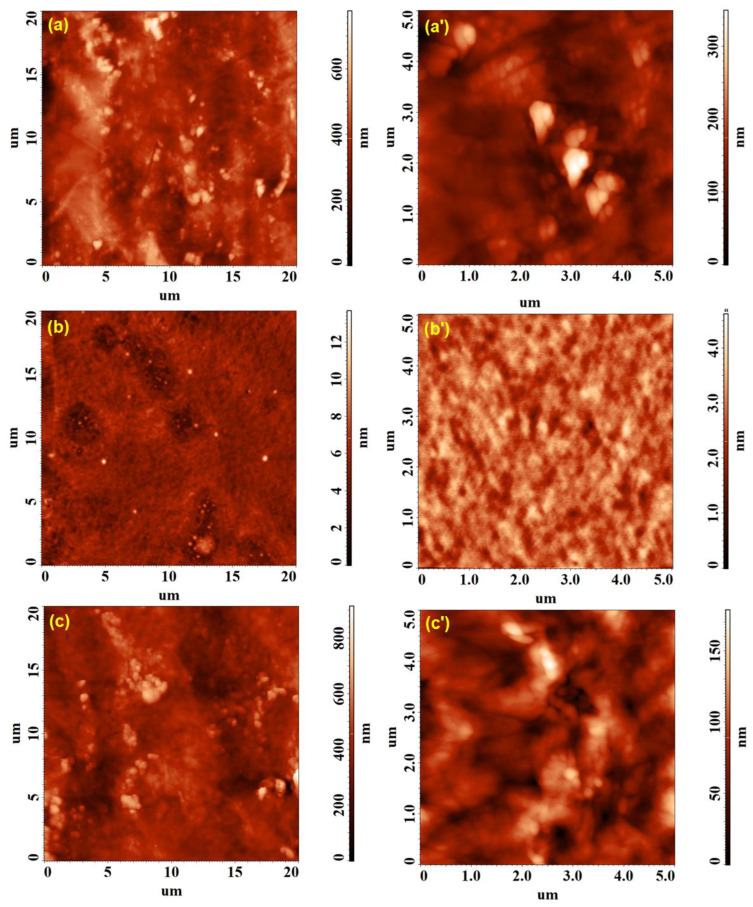
AFM topographical images collected on 20 × 20 µm^2^ and 5 × 5 µm^2^ for pristine HPMC (**a**,**a′**), PVP (**b**,**b′**) and HPMC/PVP (**c**,**c′**) samples.

**Figure 9:**
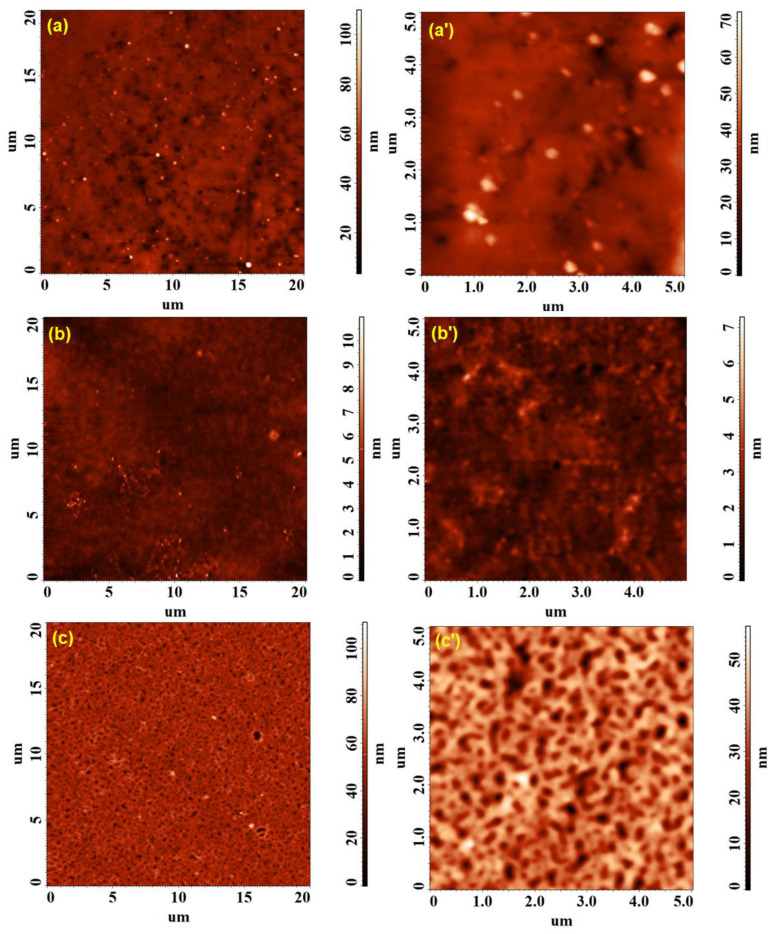
AFM topographical images collected on 20 × 20 µm^2^ and 5 × 5 µm^2^ for HPMC-AgNPs (**a**,**a′**), PVP-AgNPs (**b**,**b′**) and HPMC/PVP-AgNPs (**c**,**c′**) samples after incorporating Ag nanoparticles.

The morphological changes determined on the surface of the HPMC, PVP and HPMC/PVP samples by the incorporation of the Ag nanoparticles are presented in the topographical AFM images on large (20 × 20 µm^2^) and small (5 × 5 µm^2^) scanning areas from Figure 8 and Figure 9. Root mean square roughness (Sq), surface bearing index (Sbi) and the surface core fluid retention index (Sci) were the parameters used to quantify these modifications.

A first finding would be the fact that, for the pristine samples (Figure 8), Sq decreases with the decrease in the investigated scanning area (Table 3), due to the fact that the contribution of the morphological formations observable on the large surface was minimal during the investigations on small surfaces. On the other hand, after embedding the AgNPs in the polymers (Figure 9a–b′) and the mixture of polymers (Figure 9c,c′), very close values of Sq were found for each individual sample, both for the scanning area of 20 × 20 µm^2^ and for the 5 × 5 µm^2^, indicating the preservation of the surface uniformity at different scales. Moreover, according to the data from Table 3, the inclusion of nanoparticles leads to much smoother films, Sq decreases considerably for HPMC-AgNPs and HPMC/PVP-AgNPs and less significantly for PVP-AgNPs (given the fact that the initial roughness for PVP was more less than 1 nm).

Moreover, depending on the structure of the polymer in which the Ag nanoparticles was embedded, the morphological surface appearance of the samples was different. In the case of HPMC-AgNPs (Figure 9a,a′), the nanoparticles on the surface showed a slight tendency to overcrowd, the average diameter of the agglomerations ranging between 350 and 470 nm. For the PVP-AgNPs, the nanoparticles that apparently from AFM are individual, visible on the surface, partially embedded, from TEM, it was demonstrated that they showed a slight tendency to agglomerate, with the average diameter of the agglomerations being 130–160 nm (Figure 9b,b′). This tendency was probably induced by the presence of PVP, also in the case of the HPMC/PVP/Ag mixture. From Figure 9c,c′ it can be seen that the vast majority of nanoparticles were embedded in the material, leaving behind a rather porous material on the surface, with very few individual particles; the average diameter of the agglomerations being around 250 nm.

Following the analysis of the morphological aspect of the films, it seems that HPMC/PVP allows for the most successful incorporation of the particles, as they are also dispersed (determined by PVP) and well incorporated into the bulk. The fact that the nanoparticles are well incorporated and are not on the surface (in which case they can be easily detach from the place of fixation), can be a positive aspect. In this way, their antimicrobial activity is obviously improved and at the same time the migration of AgNps is avoided. Regarding the evaluation of the texture by means of the non-dimensional functional parameters (Table 3), it was found that the lower surface bearing (Sbi) and core fluid retention (Sci) indices calculated after adding silver denote their lower load-bearing capacity and low fluid retention ability in the core region of the relief. These characteristics are favorable for the purposed applications of the improved films, discouraging the growth of bacteria and obstructing the development of microbial colonies.

**Figure 10:**
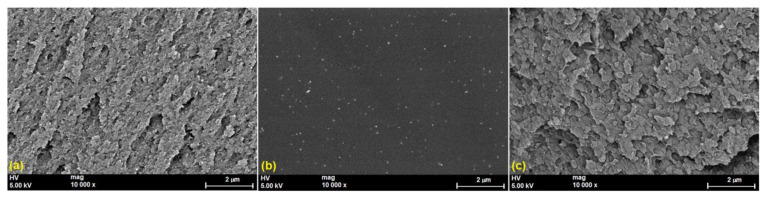
SEM images of the studied samples after incorporating Ag nanoparticles: HPMC-AgNPs (**a**), PVP-AgNPs (**b**) and HPMC/PVP-AgNPs (**c**) recorded with a magnification of 10,000×.

Additionally, the impact of PVP as a stabilizing agent, reflected by a good dispersion and capture/catching of AgNPs in the HPMC matrix, was also confirmed with the SEM images, recorded with 10,000× magnification (Figure 10). Thus, similar to the AFM data, it was visible from the SEM images that the aggregation between the particles was missing, and the particles were uniformly dispersed in the case of PVP-AgNPs (Figure 10b). In contrast, the SEM images for the HPMC-AgNPs film (Figure 10a) showed a surface with layered particles, developed on the surface of the film.

**Figure 11:**
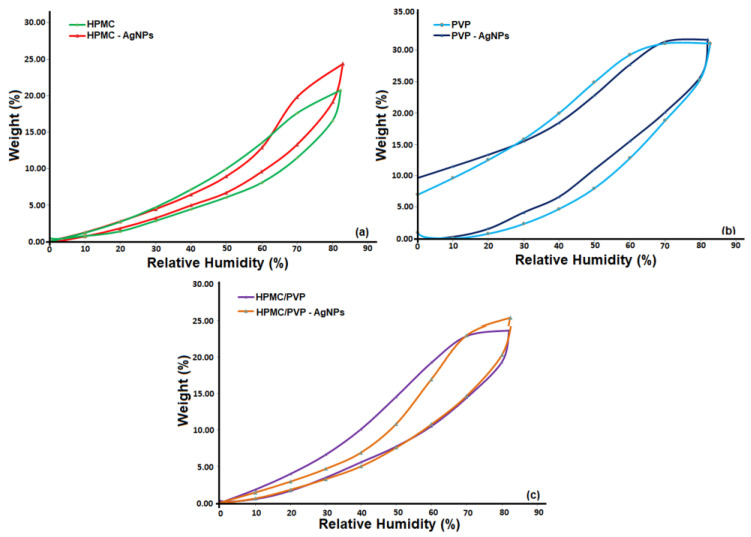
Sorption–desorption isotherms for studied materials: (**a**) HPMC and HPMC-AgNPs, (**b**) PVP and PVP-AgNPs, (**c**) HPMC/PVP and HPMC/PVP-AgNPs.

This changing tendency in the film structure as a result of the distribution of AgNPs in polymeric matrix can be evidenced through parameters obtained from the sorption-desorption curves (Figure 11). The most important surface parameters for the studied films were obtained using the Brunauer-Emmett Teller kinetic model, BET [78], described in detail in another study [7] by modeling the sorption isotherms registered under dynamic conditions (Figure 11, Table 4).

Investigations of the studied surfaces, based on water vapor sorption data, demonstrated that after the incorporation of AgNPs into polymers matrix and their blend, the sorption capacity values were very close. Furthermore, analyzing the results listed in the Table 4, it can be highlighted that the values of the specific area for both pure samples and those with incorporated silver did not change significantly. On the other hand, the difference observed between the specific surface area values for the pure samples compared to those loaded with silver can be attributed to the surface chemistry and morphological characteristics. This is a straightforward consequence of the structural characteristics of the studied materials, corroborating the results with the higher affinity to moisture of films based PVP, leading to a higher capacity of interaction with water molecules and, therefore, providing films with increased hydrophilicity.

In accordance with [77], the phenomenon of nanoparticle agglomeration occurs when they are of small sizes. This can be explained by the fact that their dispersion in the matrix is limited as result of their small dimensions, which means that the specific surface and surface energy will be higher and intermolecular forces stronger [77]. Based on the above, our findings have shown that the specific surface area values for the samples loaded with silver were lower and thus the nanoparticles were well incorporated and were not on the surface; an aspect also confirmed using the above data, regarding the morphology and distribution of the nanoparticles. Therefore, according to our results, we can conclude that the structural and surface properties of the films dictate their functionality and biological capacity.

### 3.4. Antimicrobial Activity Testing

**Figure 12:**
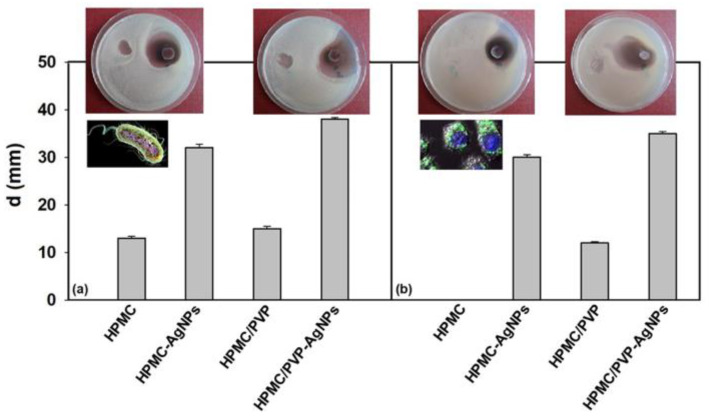
Antimicrobial activity expressed by the diameter of the inhibition zone, (d, mm) for studied materials against *Escherichia coli* (**a**) and *Staphylococcus aureus* (**b**) microorganisms. Pictures included represent the antimicrobial screening tests for evaluated materials. In each figure, the inhibition area on the right side was recorded for samples with embedded AgNPs.

To investigate the impact of the bactericidal properties and distinctive mode of action of AgNps on bioactivity of new formulation/materials based on HPMC, the microbial test was performed. Thus, the studied polymeric materials were examined for their capability of inhibiting the growth of various pathogens using the agar diffusion method. By measuring the diameter of the inhibition zones established after incubating the samples at 37 °C and 24 h, the susceptibility of the tested materials against two bacterial strains *Escherichia coli* and *Staphylococcus aureus* was specified (Figure 12).

At a first analysis it can be seen that no zone of growth inhibition was visualized for the *S. aureus*, around the HPMC film, as expected (Figure 12b). This result was in accordance with the literature, to not show any antimicrobial activity against different bacterial strains (e.g., *Listeria*, *Staphylococcus*, *Bacillus*, and *Enterococcus*) [79]. Instead, it was found that samples containing AgNPs, i.e., HPMC and the blend using PVP for stabilizing AgNPs, exhibited increased sensitivity to the tested bacteria (Figure 12). Thus, AgNPs embedded using PVP as a stabilizing agent in the HPMC matrix, i.e., the HPMC/PVP-AgNPs sample, showed a higher antibacterial activity compared to the HPMC-AgNPs. The high difference observed for antimicrobial effectiveness can be explained with the mechanism of the bactericidal effect of AgNPs against tested strains. Although the exact mechanism of action of AgNPs was not elucidated, the literature [80] mentions that the conversion of AgNPs to Ag^+^ and the generation of active oxygen and Ag^0^ could be the most likely way in which AgNPs exert their inhibitory effect on the growth of microorganisms. Thus, due to the capacity of active oxygen to diffuse into the environment, a direct interaction between the antimicrobial agent and the bacteria is not necessary. Silver ions can cause disruption of the protein structure, ultimately leading to apoptosis, as a result of their binding to nucleophilic amino acid residues in the protein structure [81,82,83]. Mechanistically, bacterial cells protect themselves by binding silver to suitable molecules like metallothioneins [84]. Thus, we can state that this dependence can be a result of a cumulative effect represented on the one hand by the ability of the particles to attach and penetrate the cell membrane, and on the other hand by the higher surface-to-volume ratio of the nanoparticles.

On the other hand, it is well documented that Gram-positive and Gram-negative bacteria differ from each other in terms of the structure of their cell membrane, namely by the thickness of the peptidoglycan layer, but also by using the presence/absence of teichoic acid [85]. Since the peptidoglycan layer is a specific characteristic of bacteria cells, then the antimicrobial activity of Ag nanoparticles must be correlated with this layer. In addition, it is also known that Gram-positive bacteria are less sensitive to the action of AgNPs than Gram-negative bacteria, probably due to the cell wall characteristics [86]. In agreement with the above, the effectiveness of Ag nanoparticles against *E. coli* was observed, which allows us to conclude that Gram-negative bacteria were more sensitive to the studied materials than Gram-positive bacteria. However, the diameter of the inhibition zones does not reflect the maximum antibacterial potential of the studied materials, because there is a small probability that all the AgNPs embedded in the polymeric films were able to interact directly with the microorganisms on the plate. These results showed the strong antimicrobial activity of the investigated materials, highlighted by the absence of microorganism growth even at the lowest silver concentration of 0.05 mg/mL for all tested bacterial strains. Therefore, the combination of the studied polymer materials (HPMC, HPMC/PVP)—silver proved to be essential in antimicrobial treatment, due to the increase in the spectrum of antimicrobial activity by using the compounds with synergistic or additive action.

## 4. Conclusions

This work represents an innovative route for the development of new polymeric materials based on inorganic active agents, HPMC/PVP-AgNPs, with a good potential for bio-applications. In this context, the formulation/design of antimicrobial materials based on HPMC with various modes of action on microorganisms through the introduction of inorganic antimicrobial agents was highlighted. This brings new benefits compared with those already presented in the literature through the effects induced by the solution parameters, structural-compositional and morphological characteristics which dictate their functionality and biological ability. Thus, the use of HPMC/PVP-AgNPs materials combines the properties of the polymers, and through the applied technique ensures the efficiency and selectivity of the obtained materials. At the same time, it was observed that the multifunctional role played by PVP to ensure an optimal composition (70/30 wt.%/wt.% composition of HPMC/PVP blend) that favors a good stabilization of silver nanoparticles (AgNPs) and thus an antimicrobial activity suitable for biomedical applications. According to the obtained results, we can conclude that the structural and surface properties of the materials dictate their functionality and biological activity. Additionally, the impact of the bactericidal properties and distinctive action mode of AgNPs on bioactivity of new HPMC-based formulations, was highlighted by the absence of microorganism growth even at low silver concentrations.

Consequently, based on the results obtained in the current study, we can state that the cellulose-bioactive materials with AgNPs present have appropriate properties for potential applications in various fields, from biomedicine to the environment. 

## Figures and Tables

**Table 1:** Flow behavior index, n, the consistency index, k, and the corresponding regression coefficients (r^2^), for HPMC/PVP systems at different mixing ratio (wt.%/wt.%).

Systems	n	k	r^2^
100/0	1.001	1.746	0.999
95/5	0.991	0.847	0.999
90/10	0.988	0.637	0.999
85/15	0.965	0.577	0.999
80/20	0.960	0.545	0.999
75/25	0.959	0.455	0.997
70/30	0.926	0.343	0.998
0/100	0.295	0.039	0.993
	0.663	0.014	0.993

**Table 2:** Parameters obtained using the TEM and DLS methods on the silver nanoparticles from the studied polymer matrices: the mean particle size (nm) and the polydispersity index (PDI).

Parameters	Sample		
	HPMC-AgNPs	PVP-AgNPs	70 HPMC/30 PVP-AgNPs
Particle size	TEM method		
	51.2 ± 1.8	20.4 ± 1.3	33.2 ± 1.6
	DLS method		
Z-average	53.09 ± 0.6	21.01 ± 0.3	36.60 ± 0.4
PDI	0.382	0.225	0.179

**Table 3:** Texture roughness parameters: root mean square roughness, (Sq), surface bearing index (Sbi), surface core fluid retention index (Sci) collected on 20 × 20 µm^2^ and 5 × 5 µm^2^ AFM images for HPMC, PVP and HPMC/PVP samples before and after incorporating AgNPs.

Sample/Scanning Area	Sq (nm)	Sbi	Sci
HPMC			
20 × 20 µm^2^ 5 × 5 µm^2^	79.4 38.8	0.304 0.257	1.689 1.714
PVP			
20 × 20 µm^2^ 5 × 5 µm^2^	0.8 0.4	0.116 0.367	1.441 1.500
HPMC/PVP			
20 × 20 µm^2^ 5 × 5 µm^2^	82.1 23.3	0.212 0.373	1.673 1.736
HPMC-AgNPs			
20 × 20 µm^2^ 5 × 5 µm^2^	6.3 6.0	0.050 0.175	1.231 1.381
PVP-AgNPs			
20 × 20 µm^2^ 5 × 5 µm^2^	0.6 0.6	0.086 0.151	1.501 1.629
HPMC/PVP-AgNPs			
20 × 20 µm^2^ 5 × 5 µm^2^	7.6 7.4	0.148 0.578	1.160 1.216

**Table 4:** Surface parameters of the studied materials evaluated based on the sorption/desorption isotherms, water vapor sorption capacity, W, and specific surface area, A, evaluated using the BET method.

Surface	W (%)		A (m^2^/g)	
	Silver Free	Silver Embedded	Silver Free	Silver Embedded
HPMC	20.78	24.40	363.02	272.54
PVP	31.14	31.74	453.74	295.65
70/30 HPMC/PVP	23.63	25.40	457.50	289.78

## Data Availability

Not applicable.

## References

[1] Spellberg B., Guidos R., Gilbert D., Bradley J., Boucher H.W., Scheld W.M., Bartlett J.G., Edwards J. (2008). The epidemic of antibiotic-resistant infections: A call to action for the medical community from the infectious diseases society of America. Clin. Infect. Dis..

[2] Gupta B., Mishra V., Gharat S., Momin M., Omri A. (2021). Cellulosic polymers for enhancing drug bioavailability in ocular drug delivery systems. Pharmaceuticals.

[3] Abdelhamid H.N., Mathew A.P. (2022). Cellulose-based nanomaterials advance biomedicine: A Review. Int. J. Mol. Sci..

[4] Filimon A., Avram E., Dunca S. (2015). Surface and interface properties of functionalized polysulfones: Cell-material interaction and antimicrobial activity. Polym. Eng. Sci..

[5] Xie Y., Qiao K., Yue L., Tang T., Zheng Y., Zhu S., Yang H., Fang Z. (2022). A self-crosslinking, double-functional group modified bacterial cellulose gel used for antibacterial and healing of infected wound. Bioact. Mater..

[6] Pan Y., Xia Q., Xiao H. (2019). Cationic polymers with tailored structures for rendering polysaccharide-based materials antimicrobial: An overview. Polymers.

[7] Filimon A., Stoica I., Onofrei M.D., Bargan A., Dunca S. (2018). Quaternized polysulfones-based blends: Surface properties and performance in life quality and environmental applications. Polym. Test..

[8] Korica M.D., Kramar A., Peršin Fratnik Z., Obradovic B., Kuraica M.M., Dojcinovic B., Fras Zemljic L., Kostic M. (2022). Obtaining medical textiles based on viscose and chitosan/zinc nanoparticles with improved antibacterial properties by using a dielectric barrier discharge. Polymers.

[9] Krzywicka A., Megiel E. (2020). Silver-polystyrene (Ag/PS) nanocomposites doped with polyvinyl alcohol (PVA)-Fabrication and bactericidal activity. Nanomaterials.

[10] Ghadermazi R., Hamdipour S., Sadeghi K., Ghadermazi R., Khosrowshahi Asl A. (2019). Effect of various additives on the properties of the films and coatings derived from hydroxypropyl methylcellulose-A review. Food Sci. Nutr..

[11] Park H.J., Weller C.L., Vergano P.J., Testin R.F. (1993). Permeability and mechanical properties of cellulose-based edible films. J. Food Sci..

[12] Qiu H., Si Z., Luo Y., Feng P., Wu X., Hou W., Zhu Y., Chan-Park M.B., Xu L., Huang D. (2020). The mechanisms and the applications of antibacterial polymers in surface modification on medical devices-A review. Front. Bioeng. Biotechnol..

[13] Olmos D., González-Benito J. (2021). Polymeric materials with antibacterial activity: A review. Polymers.

[14] Rhim J.W., Hong S.I., Park H.M., Ng P.K.W. (2006). Preparation and characterization of chitosan-based nanocomposite films with antimicrobial activity. J. Agric. Food Chem..

[15] Liu M., Bauman L., Nogueira C.L., Aucoin M.G., Anderson W.A., Zhao B. (2022). Antimicrobial polymeric composites for high-touch surfaces in healthcare applications. Curr. Opin. Biomed. Eng..

[16] Bustamante-Torres M., Arcentales-Vera B., Estrella-Nuñez J., Yánez-Vega H., Bucio E. (2022). Antimicrobial activity of composites-based on biopolymers. Macromol.

[17] Javed R., Rais F., Fatima H., Haq I.U., Kaleem M., Naz S.S., Ao Q. (2020). Chitosan encapsulated ZnO nanocomposites: Fabrication, characterization, and functionalization of bio-dental approaches. Mater. Sci. Eng. C.

[18] Hussein M.A.M., Grinholc M., Dena A.S.A., El-Sherbiny I.M., Megahed M. (2021). Boosting the antibacterial activity of chitosan–gold nanoparticles against antibiotic–resistant bacteria by Punicagranatum L. extract. Carbohydr. Polym..

[19] Chen L., Li Z., Chen M. (2019). Facile production of silver-reduced graphene oxide nanocomposite with highly effective antibacterial performance. J. Environ. Chem. Eng..

[20] Yañez-Macías R., Muñoz-Bonilla A., De Jesús-Tellez M.A., Maldonado-Textle H., Guerrero-Sánchez C., Schubert U.S., Guerrero-Santos R. (2019). Combinations of antimicrobial polymers with nanomaterials and bioactives to improve biocidal therapies. Polymers.

[21] Vasconez M.B., Flores S.K., Campos C.A., Alvarado J., Gerschenson L.N. (2009). Antimicrobial activity and physical properties of chitosan-tapioca starch based edible films and coatings. Food Res. Int..

[22] McMullen R.L., Ozkan S., Gillece T. (2022). Physicochemical properties of cellulose ethers. Cosmetics.

[23] Ozkan S., Alonso C., McMullen R. (2020). Rheological fingerprinting as an effective tool to guide development of personal care formulations. Int. J. Cosmet. Sci..

[24] Li K., Du H., Zheng T., Liu H., Zhang M., Zhang R., Li H., Xie H., Zhang X., Ma M. (2021). Recent advances in cellulose and its derivatives for oilfield applications. Carbohydr. Polym..

[25] Villalobos R., Chanona J., Hernandez P., Gutiaerrez G., Chiralt A. (2005). Gloss and transparency of hydroxypropyl methylcellulose films containing surfactants as affected by their microstructure. Food Hydrocoll..

[26] Brindle L.P., Krochta J.M. (2008). Physical properties of whey protein hydroxypropylmethylcellulose blend edible films. J. Food Sci..

[27] Burdock G.A. (2007). Safety assessment of hydroxypropyl methylcellulose as a food ingredient. Food Chem. Toxicol..

[28] Von Schantz L., Schagerlöf H., Nordberg Karlsson E., Ohlin M. (2014). Characterization of the substitution pattern of cellulose derivatives using carbohydrate-binding modules. BMC Biotechnol..

[29] Moustafa M., Fouda G., Bobbarala V. (2012). Antibacterial modification of textiles using nanotechnology. A Search for Antibacterial Agents.

[30] Rathnayake W.G.I.U., Ismail H., Baharin A., Bandara I.M.C.C.D., Rajapakse S. (2014). Enhancement of the antibacterial activity of natural rubber latex foam by the incorporation of zinc oxide nanoparticles. J. Appl. Polym. Sci..

[31] Rajendra R., Balakumar C., Ahammed H.A.M., Jayakumar S., Vaideki K., Rajesh E. (2010). Use of zinc oxide nano particles for production of antimicrobial textiles. Int. J. Eng. Sci. Technol..

[32] De Moura M.R., Mattoso L.H., Zucolotto V. (2012). Development of cellulose based bactericidal nanocomposites containing silver nanoparticles and their use as active food packaging. J. Food Eng..

[33] Dong C., Zhang X., Cai H. (2014). Green synthesis of monodisperse silver nanoparticles using hydroxy propyl methyl cellulose. J. Alloys Compd..

[34] Suwan T., Khongkhunthian S., Okonogi S. (2019). Silver nanoparticles fabricated by reducing property of cellulose derivatives. Drug Discov. Ther..

[35] Ayrancí E., Büyüktaş B.Ş., Çetin E.E. (1997). The effect of molecular weight of constituents on properties of cellulose-based edible films. LWT Food Sci. Technol..

[36] Travan A., Pelillo C., Donati I., Marsich E., Benincasa M., Scarpa T., Semeraro S., Turco G., Gennaro R., Paoletti S. (2009). Non-cytotoxic silver nanoparticle-polysaccharide nancomposites with antimicrobial activity. Biomacromolecules.

[37] Fu J., Ji J., Fan D., Shen J. (2006). Construction of antibacterial multilayer films containing nanosilver via layer-by-layer assembly of heparin and chitosan-silver ions complex. J. Biomed. Mater. Res. Part A.

[38] Sanpui P., Murugadoss A., Prasad P.V.D., Ghosh S.S., Chattopadhyay A. (2008). The antibacterial properties of a novel chitosan-Ag-nanoparticle composite. Int. J. Food Microbiol..

[39] Krasniewska K., Galus S., Gniewosz M. (2020). Biopolymers-based materials containing silver nanoparticles as active packaging for food applications—A review. Int. J. Mol. Sci..

[40] Zhang W., Ye G., Liao D., Chen X., Lu C., Nezamzadeh-Ejhieh A., Khan M.S., Liu J., Pan Y., Dai Z. (2022). Recent advances of silver-based coordination polymers on antibacterial applications. Molecules.

[41] Huang H.H., Ni X.P., Loy G.L., Chew C.H., Tan K.L., Loh F.C., Deng J.F., Xu G.Q. (1996). Photochemical formation of silver nanoparticles in poly(n-vinylpyrrolidone). Langmuir.

[42] Carotenuto G. (2001). Synthesis and characterization of poly(N-vinylpyrrolidone) filled by monodispersed silver clusters with controlled size. Appl. Organomet. Chem..

[43] Arpa M.D., Ünükür M.Z., Erim Ü.C. (2021). Formulation, characterization and in vitro release studies of terbinafine hydrochloride loaded buccal films. J. Res. Pharm..

[44] Sun Z., Zhang H., He H., Sun L., Zhang X., Wang Q., Li K., He Z. (2019). Cooperative effect of polyvinylpyrrolidone and HPMC E5 on dissolution and bioavailability of nimodipine solid dispersions and tablets. Asian J. Pharm. Sci..

[45] Józó M., Simon N., Yi L., Móczó J., Pukánszky B. (2022). Improved release of a drug with poor water solubility by using electrospun water-soluble polymers as carriers. Pharmaceutics.

[46] Aung N.N., Ngawhirunpat T., Rojanarata T., Patrojanasophon P., Opanasopit P., Pamornpathomkul B. (2020). HPMC/PVP dissolving microneedles: A promising delivery platform to promote trans-epidermal delivery of alpha-arbutin for skin lightening. AAPS PharmSciTech.

[47] Hu M., Li C., Li X., Zhou M., Sun J., Sheng F., Shi S., Lu L. (2018). Zinc oxide/silver bimetallic nanoencapsulated in PVP/PCL nanofibres for improved antibacterial activity. Artif. Cells Nanomed. Biotechnol..

[48] Ramalingam V., Varunkumar K., Ravikumar V., Rajaram R. (2018). Target delivery of doxorubicin tethered with PVP stabilized gold nanoparticles for effective treatment of lung cancer. Sci. Rep..

[49] Ramalingam V., Raja S., Harshavardhan M. (2020). In situ one-step synthesis of polymer-functionalized palladium nanoparticles: An efficient anticancer agent against breast cancer. Dalton Trans..

[50] Bryaskova R., Pencheva D., Nikolov S., Kantardjiev T. (2011). Synthesis and comparative study on the antimicrobial activity of hybrid materials based on silver nanoparticles (AgNps) stabilized by polyvinylpyrrolidone (PVP). J. Chem. Biol..

[51] Bigogno R.G., Dias M.L., Manhães N.M.B., Rodriguez R.J.S. (2022). Integrated treatment of mining dam wastewater with quaternized chitosan and pan/hpmc/agno3 nanostructured hydrophylic membranes. J. Polym. Environ..

[52] Qiu Y., Sun X., Lin X., Yi W., Jiang J. (2022). An injectable metal nanoparticle containing cellulose derivative-based hydrogels: Evaluation of antibacterial and in vitro-vivo wound healing activity in children with burn injuries. Int. Wound J..

[53] Filimon A., Onofrei M.D. (2021). New insights on solvent implications in the design of materials based on cellulose derivatives using experimental and theoretical approaches. Materials.

[54] Witten T.A., Cohen M.H. (1985). Crosslinking in shear-thickening ionomers. Macromolecules.

[55] Reiner M. (1960). Deformation, Strain and Flow.

[56] Gangopadhyay R. (2008). Exploring rheological properties of aqueous polyaniline-PVP dispersion. J. Polym. Sci. Part B Polym..

[57] Kotia A., More S., Yadav A., Mohan T.V.S.Y., Naidu A.H., Rajesh G., Sarris I.E. (2021). Rheological Properties and its effect on the lubrication mechanism of PVP K30 and PVP 40-50 G as artificial synovial fluids. Inventions.

[58] Sabzian Mellei A., Madadizadeh A., Riahi S., Kaffashi B. (2022). Synergetic effects of PVP/HEC polymers on rheology and stability of polymeric solutions for enhanced oil recovery at harsh reservoirs. J. Pet. Eng..

[59] Choi J.H., Rha C.K. (1998). Dependence of power-law parameters on polysaccharide concentration using methylan. Biotechnol. Tech..

[60] Claudel M., Schwarte J.V., Fromm K.M. (2020). New antimicrobial strategies based on metal complexes. Chemistry.

[61] Nowack B., Krug H.F., Height M. (2011). 120 Years of nanosilver history: Implications for policy makers. Environ. Sci. Technol..

[62] Xia Y., Xiong Y., Lim B., Skrabalak S.E. (2009). Shape-controlled synthesis of metal nanocrystals: Simple chemistry meets complex physics?. Angew. Chem. Int. Ed. Engl..

[63] Ni C.P., Hassan A., Kaler E.W. (2005). Structural characteristics and growth of pentagonal silver nanorods prepared by a surfactant method. Langmuir.

[64] Kittler S., Greulich C., Gebauer J.S., Diendorf J., Treuel L., Ruiz L., Gonzalez-Calbet J.M., Vallet-Regi M., Zellner R., Kcller M. (2010). The influence of proteins on the dispersability and cell-biological activity of silver nanoparticles. J. Mater. Chem..

[65] Silva E., Saraiva S.M., Miguel S.P., Correia J.I. (2014). PVP-coated silver nanoparticles showing antifungal improved activity against dermatophytes. J. Nanopart. Res..

[66] Lyutakov O., Kalachyova Y., Solovyev A., Vytykacova S., Svanda J., Siegel J., Ulbrich P., Svorcik V. (2015). One-step preparation of antimicrobial silver nanoparticles in polymer matrix. J. Nanopart. Res..

[67] Van Dong P., Ha C.H., Binh L.T., Kasbohm J. (2012). Chemical synthesis and antibacterial activity of novel-shaped silver nanoparticles. Int. Nano Lett..

[68] Zhang Z., Zhao B., Hu L. (1996). PVP protective mechanism of ultrafine silver powder synthesized by chemical reduction. Process. J. Solid State Chem..

[69] Dey A., Dasgupta A., Kumar V., Tyagi A., Verma A.K. (2015). Evaluation of the of antibacterial efficacy of polyvinylpyrrolidone (PVP) and tri-sodium citrate (TSC) silver nanoparticles. Int. Nano Lett..

[70] Kvitek O., Mutylo E., Vokata B., Ulbrich P., Fajstavr D., Reznickova A., Svorcik V. (2020). Photochemical preparation of silver colloids in hydroxypropyl methylcellulose for antibacterial materials with controlled release of silver. Coatings.

[71] El Hotabya W., Sherifa H.H.A., Hemdanb B.A., Khalilc W.A., Khalil S.K.H. (2017). Assessment of in situ-prepared polyvinylpyrrolidone-silver nanocomposite for antimicrobial applications. Acta Phys. Pol. A.

[72] Jovanovic Ž., Krklješ A., Tomic S., Miškovic-Stankovic V., Popovic S., Dragaševic M., Kacarevic-Popovic Z., Aldea A., Bârsan V. (2010). Properties of Ag/PVP Hydrogel nanocomposite synthesized in situ by gamma irradiation. Trends in Nanophysics: Theory, Experiment and Technology.

[73] Heidari B., Salmani S., Ghamsari M.S., Ahmadi M., Majles-Ara M.H. (2020). Ag/PVP nanocomposite thin film with giant optical nonlinearity. Opt. Quantum Electron..

[74] Flôr Vieira A.C., de Matos Fonseca J., Costa Menezes N.M., Monteiro A.R., Valencia G.A. (2020). Active coatings based on hydroxypropyl methylcellulose and silver nanoparticles to extend the papaya (*Carica papaya* L.) shelf life. Int. J. Biol. Macromol..

[75] Slistan-Grijalva A., Herrera-Urbina R., Rivas-Silva J.F., Avalos-Borja M., Castillon-Barraza F.F., Posada-Amarillas A. (2008). Synthesis of silver nanoparticles in a polyvinylpyrrolidone (PVP) paste, and their optical properties in a film and in ethylene glycol. Mat. Res. Bull..

[76] Kumar B., Smita K., Cumbal L., Debut A. (2017). Green synthesis of silver nanoparticles using Andean blackberry fruit extract. Saudi J. Biol. Sci..

[77] Dong Y., Zhu H., Shen Y., Zhang W., Zhang L. (2019). Antibacterial activity of silver nanoparticles of different particle size against Vibrio Natriegens. PLoS ONE.

[78] Brunauer S., Emmett P.H., Teller E. (1938). Adsorption of gases in multimolecular layers. J. Am. Chem. Soc..

[79] Imran M., El-Fahmy S., Revol-Junelles A.-M., Desobry S. (2010). Cellulose derivative based active coatings: Effects of nisin and plasticizer on physico-chemical and antimicrobial properties of hydroxypropyl methylcellulose films. Carbohydr. Polym..

[80] Ko Y.-B., Park Y.-H., MubarakAli D., Lee S.-Y., Kim J.-W. (2023). Synthesis of antibacterial hydroxypropyl methylcellulose and silver nanoparticle biocomposites via solution plasma using silver electrodes. Carbohydr. Polym..

[81] Lansdown A.B.G. (2010). Silver in Healthcare. Its Antimicrobial Efficacy and Safety in Use.

[82] Choi O., Deng K.K., Kim N.J., Ross L., Surampalli R.Y., Hu Z. (2008). The inhibitory effects of silver nanoparticles, silver ions, and silver chloride colloids on microbial growth. Water Res..

[83] Powers C.M., Badireddy A.R., Ryde I.T., Seidler F.J., Slotkin T.A. (2011). Silver Nanoparticles Compromise Neurodevelopment in PC12 Cells: Critical Contributions of Silver Ion, Particle Size, Coating, and Composition. Environ. Health Perspect..

[84] Luther E.M., Schmidt M.M., Diendorf J., Epple M., Dringen R. (2012). Upregulation of metallothioneins after exposure of cultured primary astrocytes to silver nanoparticles. Neurochem. Res..

[85] Jena P., Mohanty S., Mallick R., Jacob B., Sonawane A. (2012). Toxicity and antibacterial assessment of chitosancoated silver nanoparticles on human pathogens and macrophage cells. Int. J. Nanomed..

[86] Kim J.S., Kuk E., Yu K.N., Kim J.H., Park S.J., Lee H.J., Kim S.H., Park Y.K., Park Y.H., Hwang C.Y. (2007). Antimicrobial effects of silver nanoparticles. Nanomed. Nanotechnol. Biol. Med..

